# Diffuse large B-cell lymphoma in a patient treated with azathioprine for ulcerative colitis: a case report

**DOI:** 10.1097/MS9.0000000000000562

**Published:** 2023-04-07

**Authors:** Hari Sedai, Suraj Shrestha, Vikash Chand, Elisha Poddar, Suman Acharya, Dinesh Koirala

**Affiliations:** a Maharajgunj Medical Campus, Institute of Medicine; Departments of b Internal Medicine; c Gastroenterology and Hepatology, Tribhuvan University Teaching Hospital, Kathmandu, Nepal

**Keywords:** azathioprine, inflammatory bowel disease, lymphoma, ulcerative colitis

## Abstract

Azathioprine (AZA) used as an immunomodulator agent in the management of inflammatory bowel disease (IBD) increases the risk of the development of lymphoma.

**Case presentation:**

We present a case of a 45-year-old female receiving AZA for severe ulcerative colitis for 4 years. She presented with the chief complaints of bloody stool and abdominal pain for 1 month. Through a series of investigations including colonoscopy, contrast-enhanced computed tomography scan of the abdomen and pelvis, and biopsy with immunohistochemistry; she was diagnosed to have diffuse large B-cell lymphoma of the rectum. She is currently on a chemotherapeutic regimen and is planned for surgical resection after the completion of neoadjuvant therapy.

**Clinical discussion:**

AZA is classified as a carcinogen by the International Agency for Research on Cancer. Prolonged exposure to higher doses of AZA increases the risk of developing lymphoma in IBD. Previous meta-analysis and research indicate that the risk of development of lymphoma after the use of AZA in IBD increases by about four- to six-fold, especially in older age groups.

**Conclusions:**

AZA may increase the susceptibility to developing lymphoma in IBD, but the benefit far outweighs the risk. Precautions must be taken in prescribing AZA in older individuals which mandates periodic screening.

## Introduction

Highlights

- Azathioprine (AZA) has long been used in the treatment of inflammatory bowel disease (IBD).
- Prolonged use of AZA raises the risk of lymphoma in patients with IBD.
- Benefits of the use of AZA in IBD outweigh the risk.

IBD which encompasses Crohn’s disease and ulcerative colitis (UC) is a chronic intestinal inflammation that results from host-microbial interactions in a genetically susceptible individual. It is treated with multiple agents, including steroids, 5-aminosalicylic acid, biological agents, and immunomodulators such as AZA and 6-mercaptopurine (6-MP). In most of the population-based studies, IBD, in and of itself, does not appear to be associated with an increased risk of lymphoma^1^,^2^.

In the treatment of IBD, AZA, and 6-MP are first-line immunomodulatory agents used for the maintenance of remission at the recommended dose of 1.5–2.5 mg/kg per day^3^,^4^. AZA is effective for maintaining remission for at least 4 years, but enthusiasm for longer treatment duration is tempered by the uncertainty regarding the risk of inducing malignant disease^5^. AZA is classified as a carcinogen by the International Agency for Research on Cancer (IARC). Adverse effects serious enough to stop AZA/6-MP treatment may occur in 8–15% of IBD patients^6^.

The most common side effects of AZA/6-MP treatment are pancreatitis, allergic reactions, gastrointestinal complaints, hepatitis, and bone marrow suppression. IBD patients treated with AZA/6-MP for years are at higher risk of developing more neoplasms with a four-fold increased risk of developing lymphoma compared with the general population. The risk of lymphoma may be increased by about four-fold in patients with IBD treated with thiopurines^7^,^8^. Here, we report a case of a 45-year-old female under maintenance AZA for more than 4 years and developed lymphoma in the rectosigmoid area along with a relevant literature review. This case has been written in line with SCARE criteria^9^.

## Presentation of case

A 45-year-old female with steroid-dependent severe UC for 4 years with steroid-induced diabetes mellitus and hypertension and receiving oral AZA 125 mg, mesalazine 400 mg, prednisolone 20 mg, amlodipine 5 mg, and metformin 500 mg for the same duration presented with the complaints of bloody diarrhea and abdominal pain for 1 month and fever for 5 days. Her symptoms did not subside even on an escalating dose of steroid (prednisolone 60 mg) for which she was referred to our center.

On examination, she was ill-looking with signs of steroid toxicity with a puffy face and eyelids. She was febrile (temperature=103°F), tachycardic (pulse rate: 115 beats/min), and had a blood pressure of 100/60 mmHg. The rest of the general physical examinations were normal. Mild tenderness was present on the left iliac fossa without organomegaly. Cardiovascular, neurological, and respiratory system assessments were unremarkable.

Blood investigations revealed leukocytosis [total leukocyte count=17 400/cmm with a neutrophil predominance (90%)], low serum protein and albumin, and elevated erythrocyte sedimentation rate (40 mm/h). All other hematological and biochemical parameters were normal (Table 1) Stool examination showed plenty of pus cells per high-power field, red blood corpuscles, and undigested food particles. The stool was positive for *Clostridium difficile* toxins A, B, and glutamate dehydrogenase but no other organisms were isolated. Truelove and Witts Severity Index^10^ was calculated to determine the severity of UC, in which a score of five out of a total score of six was obtained (temperature >37.8°C, pulse rate >90/min, erythrocyte sedimentation rate >30, frequency of stool >6, and gross blood visible in stool). With the diagnosis of acute severe UC with the infectious component; oral mesalazine 400 mg, injection hydrocortisone 100 mg, injection ciprofloxacin 200 mg, injection metronidazole 500 mg, oral AZA 125 mg, oral vancomycin 500 mg, and other supportive therapies were initiated.

**Table 1:** Results showing various hematological, immunological, and biochemical parameters

	Result	Unit	Reference range
Hematology			
Total leukocyte count	17 400 (90% neutrophils)	/cmm	4000–11 000
Hb	13.4	g%	12.5-15
Platelets	281 000	/cmm	150 000–400 000
Biochemistry			
Urea	5.5	mmol/l	2.8–7.0
Creatinine	76.0	μmol/l	40–110
Sodium	141.0	mmol/l	135–146
Potassium	4.8	mmol/l	3.5–5.2
Total bilirubin	7	μmol/l	3–21
Direct bilirubin	3	μmol/l	0–5
Total protein	57	g/l	60–80
Albumin	30	g/l	37–47
A/G ratio	1.1:1	U/L	1.2–1.5
AST	11	U/L	5–40
ALT	28	U/L	5–45
ALP	78	U/L	<145.0
Gamma glutamyl transferase	21	U/L	<50.0
Uric acid	100.0	μmol/L	150–340
LDH	203	U/L	125.0–220.0
CRP test (qualitative)	Negative		
ESR	40	mm/h	0–19
CEA	0.6	ng/ml	<3.0
Carbohydrate antigen 19-9	20.7	U/ml	<37.0
Coagulation parameter and serology			
PT/INR	13/1.08	s	10–12
HIV Ab/HBsAg/HCV Ab spot/quick	Nonreactive		
Bacteriology report			
Stool C/S	No *Salmonella*/*Shigella* species isolated		
Stool routine examination/microscopic examination			
Pus cells	18-20	/HPF	
RBC	plenty	/HPF	
Undigested food particles	+++		
Parasites	Not found		
*Clostridium difficile* toxins A and B and GDH, stool			
Toxin A	Positive		Negative
Toxin B	Positive		Negative
GDH	Positive		Negative
Positive toxin A, toxin B, and GDH is positive for infection by *Clostridium difficile*			
Immunology			
CMV Ab IgM	0.27	Ratio	<0.8
CMV Ab IgG	167.25	COI	<16.0

Ab, antibody; A/G, albumin/globulin; ALP, alkaline phosphatase; ALT, alanine aminotransferase; AST, aspartate aminotransaminase; CEA, carcinoembryonic antigen; CMV, cytomegalovirus; COI, cutoff index; CRP, C-reactive protein; C/S, culture/sensitivity; ESR, erythrocyte sedimentation rate; GDH, glutamate dehydrogenas; Hb, hemoglobin; HBsAg, hepatitis B surface antigen; HCV, hepatitis C virus; Ig, immunoglobulin; LDH, lactate dehydrogenase; PT/INR, prothrombin time/international normalized ratio; RBC, red blood cell.

**Figure 1:**
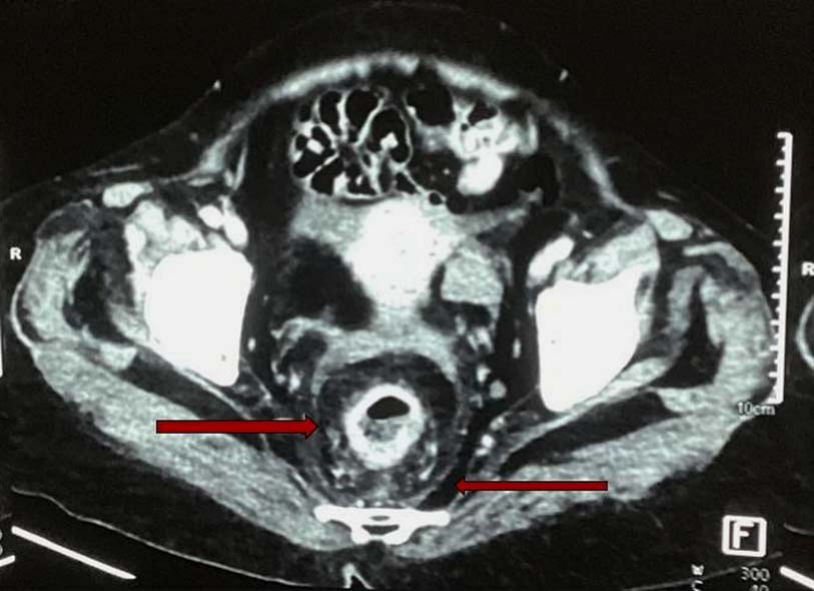
Contrast-enhanced computed tomography abdomen and pelvis showing homogeneous enhancing circumferential thickening in rectum with surrounding fat strandings (red arrows).

With unsatisfactory improvement in her symptoms over a few days, further imaging was done. Contrast-enhanced computed tomography of the abdomen and pelvis revealed a ∼3.1 cm long homogeneously enhancing circumferential thickening extending from the rectosigmoid junction up to the rectum ∼4.8 cm above the anal verge, predominantly in the posterior aspect with maximum thickness measuring ∼10.3 mm in size. Presacral space was obliterated by echogenic fat strandings; features suggestive of infective/inflammatory pathology (Fig. 1). Sigmoidoscopy revealed multiple deep ulcers in the rectum and a large circumferential ulcer with an everted margin and necrotic base in both rectum and sigmoid colon; suspicious of malignancy. Biopsy was taken from the ulcer bases and edges.

**Figure 2:**
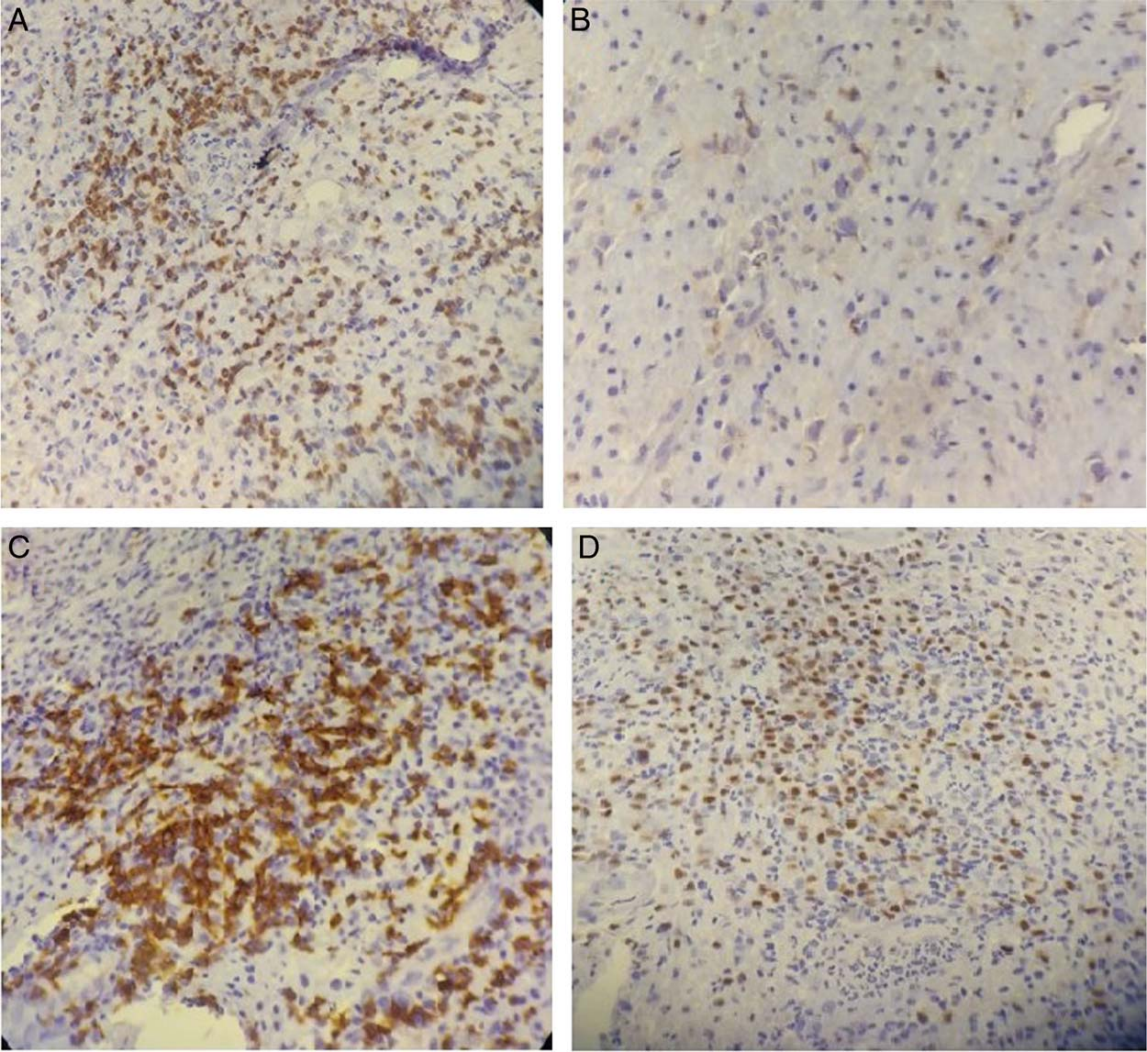
Immunohistochemistry results show tumor cells positive for CD20 (A), CD5 (B), BCL-2 (C), MUM1 (D).

A serum cytomegalovirus immunoglobulin M antibody was also sent which was within the normal range. Carcinoembryonic antigen and carbohydrate antigen 19-9 values were within their normal limit. Histopathological examination of the biopsy was consistent with UC (active phase) with suspicion of lymphoproliferative disease. From subsequent immunohistochemistry; the diagnosis of diffuse large B-cell lymphoma (DLBCL) activated B-cell type was made (Fig. 2). Bone marrow aspiration was done which was normocellular with dyserythropoietic changes and negative for malignancy. A biopsy taken from her colonic ulcer 2.5 years back was negative for dysplasia/malignancy.

The International Prognostic Index (IPI) score of the patient was calculated to be zero which indicated low-risk status and revised IPI score of zero indicating very good prognosis [age of the patient was 45 years, had normal serum lactate dehydrogenase level, Ann Arbor stage I, Eastern Cooperative Oncology Group performance status of 2 without extranodal site involvement].

AZA, a possible carcinogen, was withheld after the diagnosis of DLBCL. She is currently under R-CHOP: rituximab, cyclophosphamide, hydroxydaunorubicin (doxorubicin), vincristine (oncovin), and prednisone regimen, six cycles every 3 weeks, and planned for resection surgery for DLBCL after completion of her neoadjuvant regimen.

## Discussion

Carcinoma of the colon is a well-documented complication of UC, however, colonic lymphoma complicating UC is infrequent^11^–^13^.

Immunosuppressive therapies like AZA used in the treatment of IBD may increase the risk of lymphoma as has been demonstrated in patients with organ transplantation and other groups^14^. In addition to the effect of immunosuppression, there are specific characteristics of thiopurines such as disrupting DNA and interfering with DNA replication which may promote carcinogenesis^15^,^16^. In addition, thiopurines also directly promote DNA damage by rendering it highly sensitive to radiation and subsequent mutagenesis, particularly ultraviolet A radiation^17^–^19^. Clinical data have linked the risk of malignancy during thiopurine treatment to a total dose of AZA received^20^–^22^, thiopurine metabolite levels, and thiopurine methyltransferase mutations^23^.

Approximately 30% of all lymphomas after the use of AZA were seen in the gastrointestinal tract. Most Hodgkin lymphomas present as mediastinal masses, however, the presentation of non-Hodgkin lymphoma (NHL) is highly variable, and estimates of gastrointestinal involvement in NHL range from 5% to as much as 60%^24^. Lymphomas with IBD have were reported more commonly in the intestinal tract^25^,^26^. Although rare, there also exists a small (<1:20 000 person-years) but real chance of development of hepatosplenic T-cell lymphoma, nearly uniformly present in men younger than the age of 35 years with AZA use^24^. The increased risk of lymphoma among IBD patients is markedly lower than that observed after organ transplantation, a condition in which much greater levels of immunosuppression are achieved^27^.

The meta-analysis by Kandiel and colleagues demonstrates that there is an approximate four-fold increased risk of lymphoma in the subgroup of IBD patients treated with AZA and/or 6-MP relative as compared with the general population. The data from their meta-analysis suggest that one additional lymphoma will occur every 300–4500 years after therapy with AZA or 6-MP, depending on the age of the patient^28^. One of the meta-analyses by Kotlyar and colleagues demonstrated that patients with IBD who are taking thiopurines have a nearly six-fold higher incidence of lymphoma when compared with the general population. Their meta-analyses suggest that current thiopurine use of at least 1 year may increase the risk of lymphoma nearly six-fold. The absolute risk is highest in those over 50 years. Young males (under 30–35) may also be a high-risk population^29^. Because these data were obtained from observational studies, it is not possible to fully exclude the possibility that the increased risk of lymphoma is associated with the severity of the disease, rather than being caused by the medications^28^.

DLBCL is the most common lymphoma, accounting for about 25–30% of all NHLs. It typically presents as a rapidly growing mass or enlarging lymph nodes in a nodal or extranodal site. Though aggressive, they do respond well to six cycles of R-CHOP therapy^30^.

Although the cause of most DLBCLs remains unknown, a distinction should be made between cases arising *de novo* (referred to as primary) and those representing progression or transformation (referred to as secondary) of less aggressive lymphoma. Several chemical substances, such as pesticides and fertilizers, and medical drugs; alkylating agents used in the treatment of solid tumors and hematological malignancies; inherited immunologic deficiency diseases, and in families of patients with immunologic disorders; patients chronically immunosuppressed by drugs, particularly after organ transplants, Epstein–Barr virus, human herpesvirus 8, and HIV infections, etc. are implicated in the pathogenesis of DLBCLs^31^. The obvious risk factor for DLBCL in our case was AZA as an immunosuppressive agent.

The benefits of thiopurines, AZA, and 6-MP in maintaining remission and corticosteroid-sparing in IBD are beyond doubt^8^. In UC, when another treatment has failed, AZA may defer or avoid the need for surgery^7^. Despite growing evidence of the safety and effectiveness of immunomodulator therapy for IBD, fear of developing NHL remains one of the most common concerns among both patients and physicians in deciding whether to use these medications in the treatment of IBD^32^. Regardless, given the magnitude of the association, even if the increased risk is entirely attributable to the medications, it is unlikely to outweigh the potential benefits of these medications for most patients.

The alternatives are not without significant risk. These include uncontrolled inflammation, repeated courses of corticosteroids, alternative immunosuppressants (methotrexate and infliximab administration have also been associated with an increased risk of lymphoma), and surgery. The risk of lymphoma in IBD patients receiving thiopurines is small if there at all, but the benefits far outweigh the risks^8^.

The outcome for patients with DLBCL is typically based on risk assessment utilizing the IPI^33^. The IPI is calculated based on the following variables, with one point being assigned to each: age 60 years and older, elevated lactate dehydrogenase level, stage III–IV disease, Eastern Cooperative Oncology Group score, and more than one extranodal site of disease. The total score is tallied and patients are stratified into different risk groups as follows: low with a score of 0–1, low-intermediate with a score of 2, high-intermediate with a score of 3, and high with a score of 4–5^34^. The stage of disease as well as the IPI score is informative in the choice of therapy. The regimen for localized large B-cell lymphoma include chemoimmunotherapy for three to four cycles (CHOP) and subsequent involved-field radiation therapy^35^. The standard approach for patients with advanced-stage DLBCL is treatment with R-CHOP chemotherapy for six cycles in every 21 days^36^.

## Conclusions

AZA used as an immunomodulator therapy in the management of IBD increases the risk of the development of lymphoma but has a very rare occurrence. DLBCL is the most common lymphoma and immunosuppressive therapies like AZA may be incriminated in its pathogenesis. However, the benefits outnumber the risks, and is a better choice than its alternatives.

## Ethical approval

Not required.

## Consent for publication

Written informed consent was obtained from the patient for the publication of this case report and accompanying images. A copy of the written consent from the patient for publication is available for review by the Editor-in-Chief of this journal on request.

## Sources of funding

None.

## Author contribution

S.S., H.S., V.C., and E.P. were involved in the study concept, data collection, and writing of the manuscript. S.A. and D.K. were involved in the treatment and reviewing of the manuscript. All the authors were involved in the final review of the manuscript.

## Conflicts of interest disclosure

The authors declare that they have no financial conflict of interest with regard to the content of this report.

## Research registration unique identifying number (UIN)

Not applicable.

## Guarantor

Suraj Shrestha.

## Provenance and peer review

Not commissioned, externally peer-reviewed.

## References

[1] Lewis JD Bilker WB Brensinger C . Inflammatory bowel disease is not associated with an increased risk of lymphoma. Gastroenterology 2001;121:1080–1087. 11677199 10.1053/gast.2001.28703

[2] Bernstein CN Blanchard JF Kliewer E . Cancer risk in patients with inflammatory bowel disease: a population-based study. Cancer 2001;91:854–862. 11241255 10.1002/1097-0142(20010215)91:4<854::aid-cncr1073>3.0.co;2-z

[3] Smith MA Irving PM Marinaki AM . Review article: malignancy on thiopurine treatment with special reference to inflammatory bowel disease. Aliment Pharmacol Ther 2010;32:119–130. 20412066 10.1111/j.1365-2036.2010.04330.x

[4] Dubinsky MC . Azathioprine, 6-mercaptopurine in inflammatory bowel disease: pharmacology, efficacy, and safety. Clin Gastroenterol Hepatol 2004;2:731–743. 15354273 10.1016/s1542-3565(04)00344-1

[5] Bouhnik Y Lémann M Mary JY . Long-term follow-up of patients with Crohn’s disease treated with azathioprine or 6-mercaptopurine. Lancet 1996;347:215–219. 8551879 10.1016/s0140-6736(96)90402-x

[6] Candy S Wright J Gerber M . A controlled double blind study of azathioprine in the management of Crohn’s disease. Gut 1995;37:674–678. 8549944 10.1136/gut.37.5.674 PMC1382873

[7] Connell WR Kamm MA Dickson M . Long-term neoplasia risk after azathioprine treatment in inflammatory bowel disease. Lancet 1994;343:1249–1252. 7910274 10.1016/s0140-6736(94)92150-4

[8] McGovern DPB Jewell DP . Risks and benefits of azathioprine therapy. Gut 2005;54:1055–1059. 16009676 10.1136/gut.2004.053231 PMC1774869

[9] Agha RA Franchi T Sohrabi C . , SCARE Group. The SCARE 2020 Guideline: Updating Consensus Surgical CAse REport (SCARE) Guidelines. Int J Surg 2020;84:226–230. 33181358 10.1016/j.ijsu.2020.10.034

[10] Pabla BS Schwartz DA . Assessing severity of disease in patients with ulcerative colitis. Gastroenterol Clin North Am 2020;49:671–688. 33121688 10.1016/j.gtc.2020.08.003 PMC7510557

[11] Morowitz DA Kirsner JB . Mortality in ulcerative colitis: 1930 to 1966. Gastroenterology 1969;57:481–490. 5307491

[12] Dawson IMP Pryse-Davies J . The development of carcinoma of the large intestine in ulcerative colitis. Br J Surg 2005;47:113–128. 10.1002/bjs.18004720202 13814581

[13] Nugent FW Warren Nugent F Haggitt RC . Malignant potential of chronic ulcerative colitis. Gastroenterology 1979;76:1–5. 758130

[14] Labouyrie E Morel D Boiron JM . Peripheral T-cell lymphoma in a chronically immunosuppressed renal transplant patient. Mod Pathol 1995;8:355–359. 7567930

[15] Ling YH Chan JY Beattie KL . Consequences of 6-thioguanine incorporation into DNA on polymerase, ligase, and endonuclease reactions. Mol Pharmacol 1992;42:802–807. 1331762

[16] Somerville L Krynetski EY Krynetskaia NF . Structure and dynamics of thioguanine-modified duplex DNA. J Biol Chem 2003;278:1005–1011. 12401802 10.1074/jbc.M204243200

[17] O’Donovan P Perrett CM Zhang X . Azathioprine and UVA light generate mutagenic oxidative DNA damage. Science 2005;309:1871–1874. 16166520 10.1126/science.1114233 PMC2426755

[18] Karran P . Thiopurines, DNA damage, DNA repair and therapy-related cancer. Br Med Bull 2006;79-80:153–170. 17277075 10.1093/bmb/ldl020

[19] Kaplan HS Smith KC Tomlin P . Radiosensitization of *E. coli* by purine and pyrimidine analogues incorporated in deoxyribonucleic acid. Nature 1961;190:794–796. 13751286 10.1038/190794a0

[20] Nguyen T Vacek PM O’Neill P . Mutagenicity and potential carcinogenicity of thiopurine treatment in patients with inflammatory bowel disease. Cancer Res 2009;69:7004–7012. 19706768 10.1158/0008-5472.CAN-09-0451 PMC2749269

[21] Karran P Attard N . Thiopurines in current medical practice: molecular mechanisms and contributions to therapy-related cancer. Nat Rev Cancer 2008;8:24–36. 18097462 10.1038/nrc2292

[22] Silman AJ Petrie J Hazleman B . Lymphoproliferative cancer and other malignancy in patients with rheumatoid arthritis treated with azathioprine: a 20 year follow up study. Ann Rheum Dis 1988;47:988–992. 3207388 10.1136/ard.47.12.988 PMC1003651

[23] Thomsen JB Schrøder H Kristinsson J . Possible carcinogenic effect of 6-mercaptopurine on bone marrow stem cells. Cancer 1999;86:1080–1086. 10491537 10.1002/(sici)1097-0142(19990915)86:6<1080::aid-cncr26>3.0.co;2-5

[24] Kotlyar DS Osterman MT Diamond RH . A systematic review of factors that contribute to hepatosplenic T-cell lymphoma in patients with inflammatory bowel disease. Clin Gastroenterol Hepatol 2011;9:36–41.e1. 20888436 10.1016/j.cgh.2010.09.016

[25] Fabry TL Sachar DB Janowitz HD . Acute myelogenous leukemia in patients with ulcerative colitis. J Clin Gastroenterol 1980;2:225–227. 6935291 10.1097/00004836-198009000-00003

[26] Baker D Chiprut RO Rimer D . Colonic lymphoma in ulcerative colitis. J Clin Gastroenterol 1985;7:379–386. 3905937 10.1097/00004836-198510000-00002

[27] Opelz G Henderson R . Incidence of non-Hodgkin lymphoma in kidney and heart transplant recipients. Lancet 1993;342:1514–1516. 7902900 10.1016/s0140-6736(05)80084-4

[28] Kandiel A Fraser AG Korelitz BI . Increased risk of lymphoma among inflammatory bowel disease patients treated with azathioprine and 6-mercaptopurine. Gut 2005;54:1121–1125. 16009685 10.1136/gut.2004.049460 PMC1774897

[29] Kotlyar DS Lewis JD Beaugerie L . Risk of lymphoma in patients with inflammatory bowel disease treated with azathioprine and 6-mercaptopurine: a meta-analysis. Clin Gastroenterol Hepatol 2015;13:847–858.e4; quiz e48–e50. 24879926 10.1016/j.cgh.2014.05.015

[30] Padala SA Kallam A ■ ■ . Diffuse Large B Cell Lymphoma. In: StatPearls [Internet]. Treasure Island (FL): StatPearls Publishing; 2023. Available from: https://www.ncbi.nlm.nih.gov/books/NBK557796/ . 32491728

[31] Martelli M Ferreri AJM Agostinelli C . Diffuse Large B Cell Lymphoma. [Updated 2022 Apr 28]. In: StatPearls [Internet]. Treasure Island (FL): StatPearls Publishing; 2023 Jan. Available at: https://www.ncbi.nlm.nih.gov/books/NBK557796/.

[32] Peppercom MA . 6-mercaptopurine for the management of ulcerative colitis: a concept whose time has come. Am J Gastroenterol 1996;91:1689–1690. 8792681

[33] International Non-Hodgkin’s Lymphoma Prognostic Factors Project. A predictive model for aggressive non-Hodgkin’s lymphoma. N Engl J Med 1993;329:987–994. 8141877 10.1056/NEJM199309303291402

[34] Alamer F Alamir A Alqahtani A . Validation of the International Prognostic Index and subsequent revisions for diffuse large B-cell lymphoma in patients from the Middle East and North Africa Region. Cureus 2020;12:e9620. 32923221 10.7759/cureus.9620 PMC7478920

[35] Ansell SM . Non-Hodgkin lymphoma: diagnosis and treatment. Mayo Clin Proc 2015;90:1152–1163. 26250731 10.1016/j.mayocp.2015.04.025

[36] Coiffier B Lepage E Briere J . CHOP chemotherapy plus rituximab compared with CHOP alone in elderly patients with diffuse large-B-cell lymphoma. N Engl J Med 2002;346:235–242. 11807147 10.1056/NEJMoa011795

